# Dimensions of Oppositionality in a Brazilian Community Sample: Testing the *DSM-5* Proposal and Etiological Links

**DOI:** 10.1016/j.jaac.2013.01.004

**Published:** 2013-04

**Authors:** Fernanda Valle Krieger, Guilherme Vanoni Polanczyk, Robert Goodman, Luis Augusto Rohde, Ana Soledade Graeff-Martins, Giovanni Salum, Ary Gadelha, Pedro Pan, Daniel Stahl, Argyris Stringaris

**Affiliations:** a University of São Paulo; b Institute of Psychiatry at King’s College London; c National Institute of Developmental Psychiatry for Children and Adolescents (INCT-CNPq), Brazil; d Research Support Center on Neurodevelopment and Mental Health at the University of São Paulo; e Federal University of Rio Grande do Sul, Brazil; f Federal University of São Paulo

**Keywords:** oppositionality, dimensions, irritability, depression

## Abstract

**Objective:**

Investigating dimensions of oppositional symptoms may help to explain heterogeneity of etiology and outcomes for mental disorders across development and provide further empirical justification for the *DSM-5*–proposed modifications of oppositional defiant disorder (ODD). However, dimensions of oppositionality have not previously been tested in samples outside Europe or the United States. In this study, we used a large Brazilian community sample to compare the fit of different models for dimensions of oppositional symptoms; to examine the association of psychiatric diagnoses and symptoms with dimensions of oppositionality; and to examine the associations between dimensions of oppositionality and parental history of mental disorders.

**Method:**

A Brazilian community sample of 2,512 children 6 through 12 years old were investigated in this study. Confirmatory factorial analyses were performed to compare the fit of alternative models, followed by linear and logistic regression analyses of associations with psychiatric diagnosis and parental history of psychopathology.

**Results:**

A three-factor model with irritable, headstrong, and hurtful dimensions fitted best. The irritable dimension showed a strong association with emotional disorders in the child (*p*<.001) and history of depression (*p*<.01) and suicidality (*p*<.05) in the mother. The headstrong dimension was uniquely associated with attention-deficit/hyperactivity disorder (ADHD) in the child (*p*<.001) and with maternal history of ADHD symptoms (*p*<.05). The hurtful dimension was specifically associated with conduct disorder (*p*< .05).

**Conclusions:**

Our findings from a large community sample of Brazilian children support a distinction between dimensions of oppositionality consistent with current *DSM-5* recommendations and provide further evidence for etiological distinctions between these dimensions.

The identification of distinct dimensions of oppositional symptoms may help to explain heterogeneity of etiology and outcomes of mental disorders across development.^1^ The *DSM-5* process has recently suggested a distinction among irritable, headstrong, and hurtful dimensions in its update on oppositional defiant disorder (ODD).^2^ However, the evidence for this distinction derives exclusively from European and North American samples, and it is currently unclear whether it applies to other populations. Moreover, there are many outstanding questions about etiological distinctions between dimensions of oppositionality. To address this gap, the present study examines dimensions of oppositionality with regard to their factorial structure, psychiatric correlates, and distinct familial risk factors within a large community-based Brazilian sample of children.

Oppositional defiant disorder (ODD)—a persistent pattern of negativistic, hostile, and defiant behavior that is classified as a disruptive behavior disorder^3^—is one of the most common childhood psychiatric disorders.^4^ ODD is strongly associated with other disruptive behavior disorders, such as attention-deficit/hyperactivity disorder (ADHD) and conduct disorder (CD),^5^ but also with internalizing disorders, such as depression and anxiety.^6^ ODD often progresses to CD,^5,7,8^ but is also one of the most robust predictors of adolescent and young adult depression.^9^ Indeed, ODD is part of the developmental history of a wide range of disorders in youth and adults.^10^

Considering the heterogeneity within the ODD construct, Stringaris and Goodman^11^ provided cross-sectional and longitudinal evidence for the existence of three distinct dimensions or constructs within the ODD diagnosis: irritable, headstrong, and hurtful. The irritable dimension includes symptoms of temper outbursts, touchiness, and anger, and shows specific associations with emotional disorders. The headstrong dimension includes symptoms of arguing with grown ups, annoying others on purpose, refusing to follow rules, and blaming others for his or her own mistakes, and shows specific association with ADHD. The hurtful dimension includes symptoms of spitefulness and vindictiveness. Although all three dimensions showed cross-sectional associations with conduct problems, the hurtful dimension was differentially associated with aggressive conduct problems and callous–unemotional (CU) traits in a 3-year follow-up study.^12^

Since these findings, several groups have examined the fractionation of oppositionality into dimensions. Aebi *et al.*^13^ findings support the three-factor model initially identified by Stringaris and Goodman. Burke *et al.*^14^ found a three-factor solution with different aggregation of symptoms: a negative affective factor, including symptoms such as anger, touchiness, and spitefulness; an oppositional behavior factor, including symptoms such as temper outbursts, arguing, and defying; and an antagonistic behavior factor, including symptoms such as annoying and blaming others. Rowe *et al.* identified a two-factor solution, distinguishing between irritable and headstrong symptoms, an approach similar to the approach used by Stringaris *et al.* in a twin sample.^15,16^ In both studies, the authors were working with samples in which only one hurtful item had been measured, and included this in a broader headstrong dimension. Nevertheless, their findings confirmed that the irritable dimension was differentially strongly associated with anxiety/depression and the other dimension was strongly correlated to conduct disorder. Recently, Ezpeleta *et al.* and Wakschlag *et al.* extended findings on oppositional dimensions to pre-school samples.^17,18^

Based on this evidence, the *DSM-5* Task Force suggested a modification of the diagnosis of ODD subdividing its current eight items into three subgroups: angry/irritable mood, argumentative/defiant behavior, and vindictiveness.^2^ However, further evidence is required to examine the proposed separation. First, the question about the best model structure, that is, of the best way to carve up oppositionality, is still open. There are a number of competing models of dimensions in oppositionality, and there is no work to compare which model gives the best account of the data. Second, although the *DSM* is used internationally for research and clinical purposes, so far all studies on oppositionality and its distinct associations with psychopathology derive exclusively from European or North American populations. Neither the structure of oppositional symptoms nor its associations with other psychopathology have been tested in samples from other parts of the world. However, it is important to study samples from other cultures to determine whether the distinction between dimensions of oppositionality is a universal or a culturally restricted phenomenon and whether the proposed nosological distinctions are applicable worldwide. Third, there is a need to understand better etiological distinctions. The evidence on specific cross-sectional and longitudinal associations suggests distinct etiological underpinnings. Indeed, in a twin-based approach, Stringaris *et al.* showed evidence of genetic overlap between irritability and depression on the one hand, and between headstrong/hurtful and delinquency on the other.^15^ However, there is little further research on possible etiological distinctions. Family history is an important factor in the etiology of psychiatric disorders and disruptive behaviors are known to be highly heritable.^19^ There are associations between maternal depression and psychiatric disorders in the offspring and children of depressed mothers are at greater risk for both internalizing and externalizing disorders.^20–22^ This raises the question as to whether the dimensions could be distinguished on the basis of family history.

To address these gaps in knowledge, we used a large community sample of Brazilian children who were well characterized in terms of psychiatric diagnosis and family history, to address key issues in regard to the dimensions of oppositionality. Our aims were as follows: first, to compare the fit of different models that have been previously reported in the literature for distinguishing between dimensions of oppositional symptoms. For this purpose, we compared a one-factor structure (all ODD items forming one dimension, as originally proposed by the *DSM-IV*) versus a two-factor structure (irritable and headstrong/hurtful) versus the three-factor structure proposed by Stringaris and also by the *DSM-5* Working Group (irritable, headstrong, and hurtful) versus the three-factor structure proposed by Burke *et al.* (negative affect, oppositional behavior, and antagonistic behavior). Our second goal was to examine the pattern of associations between the dimensions of the best-fitting model and psychiatric diagnosis and symptoms. Our third goal was to examine the associations between dimensions of oppositionality and parental history of mental disorders. Our hypotheses were: The best fitting model would be the three-factor model with irritable, headstrong, and hurtful dimensions; second, associations would be detected between the irritable/negative affective dimension and emotional disorders, between the headstrong/oppositional behavioral dimension and ADHD, and between the hurtful dimension and conduct disorder; and third, associations would be detected between the irritable/negative affective dimensions and a family history of depression; between headstrong/oppositional behavior and a parental history of ADHD symptoms; and between the hurtful dimension and a parental history of antisociality and incarceration.

## Method

### Sample

This report is part of a large, community, school-based study that combines standardized evaluation from a psychiatric and cognitive neuroscience perspective, as well as genetics and neuroimaging, to inform preventive strategies in developmental psychiatry. It was performed in multiple steps involving several evaluation teams and research protocols as previously described.^23^ These steps, described here briefly, included screening, psychiatric assessments, and cognitive evaluation. Our study population in the screening phase was composed of students from public schools located close to the research centers in Porto Alegre and São Paulo, Brazil. A total of 57 schools from the two cities (22 schools in Porto Alegre and 35 schools in São Paulo) participated in the screening and enrolment procedures. Eligible subjects were those with the following characteristics: registered for school by a biological parent capable of providing consent and information about the child’s behavior; 6 to 12 years of age; and having remained in the same school during the year for logistic reasons. For screening, 9,937 informant interviews based on the Family History Survey (FHS)^24^ were conducted (involving the child’s biological mother in 88% of the families). From this pool, we selected two subgroups: a random and a high-risk stratum. For subjects in the random-selection stratum, a simple randomization procedure from school directories was used, without replacement of unavailable subjects. Selection for the high-risk stratum involved a risk-prioritization procedure, focused on individuals with a family history of a disorder and/or ongoing symptoms in one of the five targeted domains (attention-deficit/hyperactivity disorder [ADHD], anxiety, obsessive-compulsive disorder [OCD], psychosis, and learning disorders), as detected during screening. Subjects in this second high-risk stratum were oversampled and replaced by the next subject listed in the sampling frame of higher risk if not available. From 1,315 children selected in the first random stratum, 958 (73%) completed the household evaluation. There were no differences in gender, number of siblings, or presence of depressive symptoms in mothers between those who did and those who did not participate in the survey; those who participated in the study were younger (entered: 8.6 years; did not enter: 9.1 years; *p*<.001). From the 2,050 children selected for the high-risk stratum, 1,554 (76%) participated in the study. There were no differences in age, gender, number of siblings, or depressive symptoms in mothers between those who did and those who did not participate. The total sample was a combination of the random and the high-risk samples and included 2,512 subjects. Written consent was obtained from all parents of participants, and verbal assent was obtained from all children. When appropriate, written assent was also obtained. The study was approved by the ethics committee of the University of São Paulo (IORG0004884, project IRB registration number: 1132/08).

### Measures

*Strengths and Difficulties Questionnaire (SDQ)*. This is a 25-item questionnaire with robust psychometric properties. It comprises five factors, each one with five items: emotional problems, hyperactivity, conduct problems, peer problems, and prosociality.^25^ The reliability and validity of SDQ make it a good measure of the adjustment of psychopathology of children and adolescents.^26^ Although there are also youth and teacher SDQ versions, only the parental version was used on the study for logistic reasons. Here, we tested the SDQ scores of 2,512 children as dependent variables for association with the oppositional dimensions as predictors.

**FIGURE S1:**
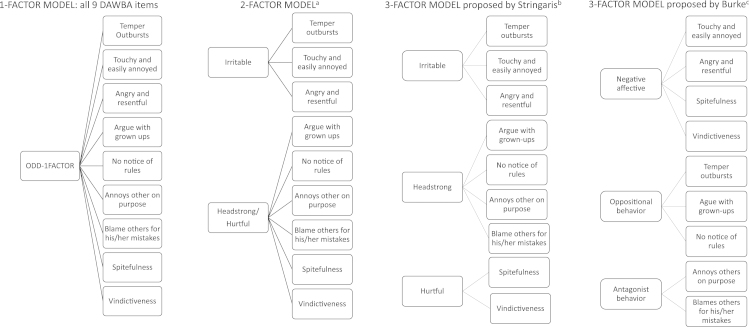
Models of oppositionality. Note: DAWBA = Development and Well-Being Assessment; ODD = oppositional defiant disorder. ^a^Model proposed by Rowe *et al.*^1^^b^Model proposed by Stringaris *et al.*^2^^c^Model proposed by Burke *et al.*^3^

*Development and Well-Being Assessment (DAWBA).* This is a structured interview administered by lay interviewers who also recorded the verbatim accounts of any reported problems.^27,28^ The questions are closely related to *DSM-IV* and ICD-10 diagnostic criteria and focus on current problems. The DAWBA makes use of skip rules that allow interviewers to omit many of the detailed questions in a section when the preliminary answer indicates a very low probability of disorder in that domain. In the case of oppositional defiant disorder (ODD), the detailed questions are skipped if both of the following conditions are met: the behavior of the child is not reported to be more troublesome than that of other children at the same age, and the conduct problems score on the SDQ is in the normal range. In the present study, 50.7% (1,274) of the sample were assessed for ODD. When the two “skip” conditions are not met, the DAWBA asks nine questions on ODD, one question on each of the 7 first *DSM-IV* items and the 2 separate questions for the “spiteful and vindictive” item. The response categories were “no more than others” (0), “a little more than others” (1), and “a lot more then others” (2). To create dimensions of oppositionality, we grouped the DAWBA’s ODD items in the four following models: first, a one-factor model, in which all nine items were loaded; second, a two-factor model: irritable and headstrong/hurtful model, using three items for irritable (“had temper outbursts?” “been touchy and easily annoyed?” “been angry and resentful?”) and six items for headstrong/hurtful (“argued with grown-ups?” “taken no notice of rules, or refused to do as s/he is told?” “seemed to do things to annoy other people on purpose?” “blamed others for his/her own mistakes?” “been spiteful?” “been vindictive?”); third, a three-factor model following Stringaris *et al.*: an irritable, headstrong, and hurtful model, preserving the irritable with three items as above (“had temper outbursts?” “been touchy and easily annoyed?” “been angry and resentful?”), headstrong with four items (“argued with grown ups?” “taken no notice of rules, or refused to do as s/he is told?” “seemed to do things to annoy other people on purpose?” “blamed others for his/her own mistakes?”), and two items for hurtful (“been spiteful?” “been vindictive?”); and fourth, a three-factor model following Burke *et al*.: negative affective, oppositional behavior and antagonistic behavior model, using four items for negative affective dimension (“been angry and resentful?” “been spiteful?” “been vindictive?” “been touchy and easily annoyed?”), three items for oppositional behavior (“had temper outbursts?” “argued with grown-ups?” “taken no notice of rules, or refused to do as s/he is told?”), and two items for antagonist behavior (“seemed to do things to annoy other people on purpose?” “blamed others for his/her own mistakes?”) (see Figure S1, available online).

*Mini-International Neuropsychiatric Interview (M.I.N.I.)*. This is a short, structured diagnostic interview, for *DSM-IV* and ICD-10 psychiatric disorders in adults. The M.I.N.I. has acceptably high validation and reliability scores.^29^ In the present study, the M.I.N.I. was administered during the household evaluation to biological mothers in 91.5% of cases. For the analyses presented in this article, we considered a family history of depression, suicidality, childhood symptoms of ADHD, and drinking as dependent variables, examining how far they were predicted by the oppositional dimensions.

*Family History Screen (FHS).* This is a screening instrument that was used for parental history of incarceration and childhood conduct symptoms as dependent variables for association with the oppositional dimensions as predictors.

### Participants With Data on Dimensions of Oppositionality

There were 2,512 children for whom there were available clinical and demographic data (see above). The characteristics of the oppositional dimensions subsample (“dimensions subsample,” i.e., those who responded affirmatively to the DAWBA’s ODD skip question, n = 1,274) and the non-dimensions subsample (n = 1,238) are summarized on Table 1. There were no differences in age, gender, or maternal education between the two groups. The oppositional dimensions subsample was more likely to come from low and very low socioeconomic backgrounds (dimensions: 16.5%; non-dimensions: 12.5%; *p* = .004) and presented significant more “any psychiatric diagnosis” (dimensions: 40.2%; non-dimension: 11.2%; *p*<.001), ODD (dimensions: 10.2%; non-dimension: 0; *p*<.001), CD (dimensions: 3.1%; non-dimensions: 0; *p*<.001), ADHD (dimensions: 19.3%; non-dimensions: 2.2%; *p*<.001) and emotional disorders (dimensions, 18.6%; nondimensions, 7.8%; *p*<.001). The differences listed above were expected, because the oppositional dimensions subsample was composed by those individuals who passed through the DAWBA’s skip rule for ODD, thus increasing their likelihood of having a disruptive and/or an emotional diagnosis.

### Statistical Analysis

Confirmatory factorial analyses (CFA) were used to compare the goodness-of-fit between the following ODD models separately: one factor; two factors (irritable, headstrong/hurtful), three factors (irritable, headstrong, and hurtful; negative affective, oppositional behavioral, and antagonist behavior). Goodness-of-fit was assessed in all models using Tucker Lewis Index (TLI), Comparative Fit Index (CFI), root mean square error of approximation (RMSEA). For CFI and TLI, values greater than 0.95 are preferred, and values near 0.90 are considered acceptable. For RMSEA, values of 0.05 or less are preferred, and values up to 0.08 are considered acceptable.^30^ We compared models using the Akaike Information Criteria (AIC) and the Bayesian Information Criteria (BIC), in which lower values indicate better fit.^31^ The difference between the two models was calculated as the ΔAIC and ΔBIC. These are calculated as the difference between a candidate model and the best (i.e., lowest AIC or BIC model); as an example, in the comparison between the one- and two-factor models, we calculate as follows: ΔΑIC = AIC_one factor_−AIC_two factors_. Values of ΔΑIC or ΔBIC equal to or greater than 10 indicate overwhelming support for the lower AIC and BIC models.^32^

In addition, χ^2^ difference tests (Δχ^2^) were conducted to compare the nested models of interest (one-factor model, two-factor [model irritable, headstrong/hurtful], and three-factor model [irritable, headstrong, and hurtful]). Those difference tests were also conducted using robust maximum likelihood and weighted least-square estimation, which showed the same pattern of results (available upon request). As the three-factor model (irritable, headstrong, and hurtful) and the three-factor model (negative affective, oppositional, and antagonistic behavior) were not nested in each other, they were compared using AIC and BIC. Analysis were performed with MPlus version 5.^33^

Multivariate logistic and linear regression analyses were conducted to test the association between family history of psychopathology, comorbidities, and SDQ scores and dimensions of oppositionality. The variables corresponding to oppositional dimensions were created based on sums of scores on DAWBA’s ODD items (0 = “no more than others, 1 = “a little more than others” and 2 = “a lot more then others”) divided by the number of items for each dimension. Multivariate linear regression models were run for the SDQ outcomes (with the three dimensions as independent variables, controlled for age and gender), and multivariate logistic regression models were run for the disorder outcomes (with the three dimensions as independent variables, controlled for age and gender); standardized coefficients and odds ratios are reported for the linear and logistic models, respectively. To compare the size of coefficients of the dependent variables that entered the final model, we used a z test (post-estimation function lincom). This tests the null hypothesis that the two coefficients are equal. An alpha level of 0.05, two-tailed, was considered significant. Analysis were performed with Stata version 10.^34^

## Results

Our first aim was to compare the goodness-of-fit of the four competing models for the oppositional dimensions through confirmatory factor analysis. The results of confirmatory factorial analysis for each model are displayed in Table 2. The upper part of Table 2 presents the goodness-of-fit parameters for each model. The three-factor model (irritable, headstrong, and hurtful) fitted the data best on all parameters: values closest to 0.95 CFI and TLI; values closest to 0.05 for RMSEA; and the largest AIC and BIC. The bottom part of Table 2 displays the results for the comparisons between models. The three-factor model structured by irritable, headstrong and hurtful was superior to the one-factor (ΔAIC 583.765, ΔBIC 237.216, and χ^2^ difference test 244.367, 1 df, *p*<.001) and two-factor models (ΔAIC 361.398, ΔBIC 331.099, and χ^2^ difference test 345.398, 2 df, *p*<0.001). The three-factor model comprising negative affective, oppositional, and antagonistic behavior was significantly better than the one-factor model (ΔAIC 78.565, ΔBIC 63.114, and χ^2^ difference test 84.565, 3 df, *p*<0.001), however, the three-factor model comprising negative affective, oppositional, and antagonistic behavior performed worse than the two-factor model irritable, headstrong/hurtful (ΔAIC –163.803, ΔBIC –174.102). Finally the two three-factor models were compared, and the one structured by irritable, headstrong, and hurtful best fitted the data (ΔAIC 505.2, ΔBIC 505.201).

The correlations among the three dimensions were as follows: irritable and headstrong, r = 0.73; irritable and hurtful, r = 0.53; and headstrong and hurtful, r = 0.59. Correlations were all highly significant (*p*< .001).

Our second aim was to adopt the best-fitting model and to examine the differential associations of each dimension with SDQ scores and psychopathology. Table 3 displays the results from linear regression within the oppositional dimensions as independent variables and SDQ scores as dependent variables. The irritable dimension showed a strong association with emotional problems (*p*<.001), and the coefficient of association for irritable was significantly higher compared to those for headstrong and hurtful. Conversely, the headstrong dimension was uniquely associated with hyperactivity (*p*<.001), and the coefficient of association for headstrong was significantly higher compared to irritable and hurtful. Headstrong and hurtful dimensions were equally associated to conduct problems. For prosociality, both headstrong and hurtful dimensions showed a negative significant association.

Using the oppositional dimensions as independent variables and psychiatric disorders as the outcome, the irritable dimension was uniquely associated with emotional disorders (*p*<.001), any anxiety disorder (*p*<.001), and major depression (*p*<.001), and the odds ratio difference confirmed the finding, with only irritable dimension index being significantly different from the other two. The hurtful dimension showed a significant association with conduct disorder (*p*<.05), but the odds ratio differences with headstrong and irritable dimensions were not significant. As predicted, the headstrong dimension showed a strong association with ADHD (*p*<.001), which was also confirmed by the odds ratio difference, showing a significant difference between headstrong and the other two dimensions. These results are displayed in Table 4.

Our third aim was to examine the association of oppositional dimensions with parental history for individual disorders (Table 5). The irritable dimension showed a specific association with family history of depression (*p*<.01) and suicidality (*p*<.05), whereas headstrong was uniquely associated with family history of childhood ADHD symptoms (*p*<.05). The three ODD dimensions were not associated with family history of drinking, imprisonment, or childhood conduct symptoms.

We repeated all analyses for each stratum (randomized and high-risk) and the results showed the same pattern of results. In particular, the irritable dimension showed strong association with emotional SDQ scores and diagnosis of depression; the headstrong dimension showed an association with ADHD diagnosis; whereas the hurtful dimension showed an association with conduct disorder. The only exception was the association between dimensions and family history; although the odds ratios suggested an association, this was not significant, which may be attributable to limited power after splitting of the sample.

## Discussion

This study provides new empirical evidence from a large Brazilian sample on oppositional dimensions model structure and associations with other mental disorders. In particular, the results show the following: first, the model distinguishing among the three dimensions of irritable, headstrong, and hurtful behaviors best fits the data; second, there are distinct associations between each of these three dimensions and other psychiatric disorders, consistent with previous studies from European and US samples; and third, the oppositional dimensions show a distinct association with family risks, in keeping with hypotheses about distinct etiological processes for each of the dimensions.

The first aim of this article was to compare models that distinguish between dimensions of oppositionality. We found that the model containing irritable, headstrong, and hurtful dimensions, as initially identified by Stringaris *et al.,*^11^ fit the data best compared with the two-factor model (irritable, headstrong/hurtful) and the three-factor model proposed by Burke *et al.*, (negative affective, oppositional behavioral, and antagonistic behavior),^14^ as indicated by comparative and absolute fit indices. These results support the current division of oppositional dimensions suggested by the *DSM-5* Taskforce in their proposal.

To our knowledge, this is the first study to test the dimensional structure of oppositionality through confirmatory factorial analysis, outside Europe and the United States. In our Brazilian sample, we found a structure similar to those suggested in previous studies,^11,12^ with distinct associations for each of the dimensions. As previously shown, the irritable dimension was strongly correlated with emotional symptoms and emotional disorders; headstrong was strongly associated with SDQ hyperactivity scores and a diagnosis of ADHD, whereas hurtful showed a strong association either with conduct symptoms on SDQ or conduct disorder. This constellation of findings suggests that dimensions of oppositionality may not be a phenomenon restricted to a particular geographic location or cultural background. Previous studies have suggested that differences in the prevalence or pattern of symptoms between countries may be due to methodological factors rather than geographic or cultural factors.^35,36^ In any case, it is important to examine for overlaps and differences in the constellation of psychiatric symptoms and disorders outside the United States or Europe, not least because 90% of children’s world population comes from low- and middle-income countries. As the *DSM* is being used worldwide, it is reassuring to find that the proposed changes for oppositionality may apply outside the United States.^37^

Our third aim was to examine specific associations between dimensions of oppositionality and parental history of psychopathology. We found a differential association between the irritable dimension and a family history of depression and suicidality. This is in keeping with previous findings concerning shared genetic risk factors between irritability and depression.^15^^,^^38^ It also demonstrates the close links between irritability and suicidal behaviors, as indicated in previous research.^39^ It is unclear what the mechanisms of shared risks between irritability and depression are. It is possible that altered affect regulation processes play a key role. For example, a reduced volume of the right dorso-lateral prefrontal cortex (DLPFC)^40,41^—an area implicated in behavior and affective processing—is found both in children with severe irritability as well as in the offspring of mothers with depression. In keeping with our expectations, we found that childhood headstrong behavior shares familial risks with parental history of ADHD symptoms during childhood. Little is known at present about what the shared mechanisms between headstrong and ADHD could be. It is possible that higher levels of cognitive impulsivity are common to both presentations, and further research is required to test this assumption. The hurtful dimension showed a specific association with conduct disorder and SDQ conduct scores. However, contrary to our prediction, we did not find an association between the hurtful dimension and a family history of imprisonment and antisocial behavior, although a true association might have been masked by the low overall prevalence of imprisonment or by selective under-reporting of socially undesirable outcomes. Future studies that will include callous and unemotional traits as an outcome might be more informative in this regard.^42^

Our study should be seen in the light of its limitations. First, the sample is not representative of the full ethnic diversity of the Brazilian population, so its results may not generalize to all sectors of society. In addition, the majority of those in the ODD dimensions subsample (those who screened positively) were from the high-risk sample, and this may limit the generalizability of our findings. Second, the evaluation was performed with only one of the parents and did not include other reporting sources. Third, the family history information was gathered retrospectively and is therefore liable to recall bias (although there is no obvious reason why this could have had differential effects on the three dimensions). Fourth, the reader should note that the hurtful model—which has only two indicators—would be under-identified in a model without any other latent variables.^43^ Future instruments should test models with more item indicators for this dimension, to increase the reliability of the latent trait model. Finally, at this point in time, our data are cross-sectional, limiting the strength of causal inferences; future studies using longitudinal data should re-examine the hypotheses tested here, possibly by using a structural equation modeling framework.

Our study has implications for researchers and clinicians. Our findings lend empirical support for a nosological model for *DSM-5* and other classifications. For research, our data extend previous findings on oppositional dimensions by identifier distinct familial associations. Clinicians may need to particularly assess one or another type of clinical correlates and possibly develop differentiated treatments according to the relative balance of irritable, headstrong, and hurtful dimensions.

In summary, our findings from a large community sample of Brazilian children support a distinction between dimensions of oppositionality, and provide further evidence for etiological distinction between dimensions.

**Clinical Guidance**

• Youth with oppositional defiant disorder (ODD) have a higher risk for a wide range of other psychiatric problems, including antisocial outcomes, attention-deficit/hyperactivity disorder (ADHD) as well as depression.
• Three different dimensions within oppositionality, termed irritable, headstrong, and hurtful, explain why there is such a wide range of ODD outcomes.
• Here we show in an independent sample from Brazil how these dimensions are differentially associated with psychiatric outcomes: irritable showed a differentially strong link with emotional and depressive disorders; headstrong with ADHD and hurtful with conduct disorder.
• In addition, we show that these different associations may be explained by family history: irritable is differentially linked to maternal depression; and headstrong with parental history of ADHD symptoms.
• Our findings lend further support for the *DSM-5* decision to distinguish between three dimensions of oppositionality in youth.

## Figures and Tables

**TABLE 1:** Sample Characteristics

	**n**	**Age, y, Mean (SD)**	**Male n (%)**	**Socioeconomic Status n (%)**			**Maternal Education: Completed High School n (%)**	**Any Diagnosis n (%)**	**ODD n (%)**	**CD n (%)**	**ADHD n (%)**	**Emotional Disorders n (%)**
**Low–Very Low**	**Medium**	**High**
Dimensions	1274	9.67 (1.9)	694 (54.4)	211 (16.5)	940 (73.7)	123 (9.6)	503 (39.9)	513 (40.2)	131 (10.2)	40 (3.1)	246 (19.3)	238 (18.6)
Non-dimensions	1238	9.73 (1.9)	641 (51.7)	155 (12.5)	927 (74.8)	515 (42.0)	515 (42.0)	139 (11.2)	0[#40]?>	0[#40]?>	28 (2.2)	97 (7.8)
	2512	NS	NS	*p* = .004	NS	*p* = .01	NS	*p*<.0001	*p*<.0001	*p*<.0001	*p*<.0001	*p*<.0001

Note: Top row of table represents the oppositional dimensions subsample, i.e. those who passed through the Development and Well-Being Assessment (DAWBA)’s skip rule for ODD. Bottom row represents non-dimension subsample those who did not pass the DAWBA’s skip rule for ODD. ADHD = attention-deficit/hyperactivity disorder; CD = conduct disorder; NS = not significant; ODD = oppositional defiant disorder.

**TABLE 2:** Confirmatory Factorial Analysis for Four Different Models of Oppositional Dimensions

	**Models of Oppositional Dimensions**							
**Maximum Likelihood**	**One-factor (All nine DAWBA Items)**	**Two-factor Irritable, Headstrong/Hurtful**^a^			**Three-factor Irritable, Headstrong, and Hurtful**^b^			**Three-factor Negative Affective, Oppositional, and Antagonistic Behavior**^c^
χ^2^	723.705	479.338			133.940			639.140
Degrees of freedom	27	26			24			24
AIC	16715.451	16473.084			16131.686			16636.886
BIC	16854.498	16617.282			16286.183			16791.384
CFI	0.887	0.926			0.982			0.900
TLI	0.849	0.898			0.973			0.850
RMSEA	0.142	0.117			0.060			0.142

**Comparisons**			**ΔAIC**^d^	**ΔBIC**^d^		**χ**^**2**^**Difference**		*p*	
1F vs. 2F			252.267	237.216		244.367(1d.f)		<.001	2-factor model better than 1-factor model
1F vs. 3F (irritable, headstrong and hurtful)			583.765	568.315		589.785(3d.f.)		<.001	3-factor__irritable, headstrong and hurtful_ better than 1-factor model
2F vs. 3F (irritable, headstrong and hurtful)			361.398	331.099		345.398(2d.f.)		<.001	3-factor__irritable, headstrong and hurtful_ better than 2-factor model
1F vs. 3F (negative affective, oppositional behavior and antagonistic behavior)			78.565	63.114		84.565(3d.f.)		<.001	3-factor __negative affective, oppositional and antagonistic behavior_ better than 1-factor
2F vs. 3F (negative affective, oppositional and antagonistic behavior)			−163.802	−174.102		Nonnested models			2 factor model better than 3-factor__negative affective, oppositional and antagonistic behavior_
3F (negative affective, oppositional and antagonistic behavior) vs. 3F (irritable, headstrong and hurtful)			505.2	505.201		Nonnested models			3-factor_ _irritable, headstrong and hurtful_ better than 3-factor__negative affective, oppositional and antagonistic behavior_

Note: 1F = one-factor model; 2F = two-factor model; 3F = three-factor model; AIC = Akaike Information Criteria; BIC = Bayesian Information Criteria; CFI = Comparative Fit Index; DAWBA = Development and Well-Being Assessment; RMSEA = root mean square error of approximation; TLI = Tucker-Lewis Index.

a Model proposed by Rowe *et al.*^7^

b Model proposed by Stringaris *et al.*^11^

c Model proposed by Burke *et al.*^14^

d ΔAIC and ΔBIC were calculated as the difference between a candidate model and the best (i.e., lowest AIC or BIC model). Values equal to or greater than 10 for ΔAIC and ΔBIC indicate overwhelming support for the lower AIC and BIC models.

**TABLE 3:** Oppositional Dimensions and Their Associations With the Strengths and Difficulties Questionnaire (SDQ)

Oppositional dimensions	**SDQ**				
**Emotional Problems**	**Hyperactivity**	**Conduct Problems**	**Peer Problems**	**Prosociality**
Irritable	0.73 (0.52, 0.94)⁎⁎⁎	0.03 (−0.17, 0.24)	−0.01 (−0.14, 0.12)	0.08 (−0.09, 0.26)	0.05 (−0.10, 0.21)
Headstrong	0.04 (−0.17, 0.26)	0.86 (0.64, 1.08)⁎⁎⁎	0.62 (0.48, 0.77)⁎⁎⁎	0.28 (0.09, 0.47)⁎⁎	−0.38 (−0.54,−0.21)⁎⁎⁎
Hurtful	0.03 (−0.13, 0.21)	−0.009 (−0.18, 0.16)	0.32 (0.21, 44)⁎⁎⁎	0.26 (0.11, 0.42)⁎⁎	−0.19 (−0.32,−0.06)⁎⁎
Comparisons	Irr Head Hurt	Head Irr Hurt	Irr Head Hurt	Irr Head Hurt	Irr Head Hurt

Note: Values correspond to standardized coefficients of multivariate linear regression models (with the three dimensions as independent variables controlled for age and gender) and their 95% confidence interval in parentheses for the oppositional dimensions. Bottom row shows comparison between individual coefficients for each oppositional dimension of symptoms, testing the null hypothesis that they not differ. Coefficients underlined are not significantly different at the level of *p*<.05. Head = headstrong; Hurt = hurtful; Irr = irritable.

⁎⁎*p* ≤.01; ⁎⁎⁎*p*≤ .001.

**TABLE 4:** Oppositional Dimensions and Their Associations With Psychiatric Diagnosis

Oppositional dimensions	**Psychiatric Diagnosis**				
**Emotional Disorders**	**Any anxiety Disorder**	**Major Depression**	**Conduct Disorder**	**ADHD**
Irritable	1.9 (1.5, 2.3)⁎⁎⁎	1.6 (1.3, 2.0)⁎⁎⁎	2.7 (1.8, 4.1)⁎⁎⁎	1.3 (0.84, 2.3)	0.94 (0.76, 1.1)
Headstrong	0.97 (0.78, 1.2)	1.0 (0.79, 1.2)	0.89 (0.61, 1.3)	1.5 (0.97, 2.5)	2.6 (2.0, 3.2)⁎⁎⁎
Hurtful	1.0 (0.88, 1.2)	0.96 (0.81, 1.1)	1.1 (0.94, 1.4)	1.3 (1.0, 1.8)⁎	1.0 (0.90, 1.2)
Comparisons	Irr Head Hurt	Irr Head Hurt	Irr Head Hurt	Irr Head Hurt	Head Irr Hurt

Note: Values correspond to standardized odds ratios of multivariate logistic regression models (with the three dimensions as independent variables controlled for age and gender) and their 95% confidence interval in brackets for the oppositional dimensions. The comparison column shows contrasts between individual odds ratios, testing the null hypothesis that they do not differ, and those with common underlining are not significantly different at the level of *p*<.05. Emotional disorders includes both depressive and anxiety disorders. ADHD = attention-deficit/hyperactivity disorder; Head = headstrong; Hurt = hurtful; Irr = irritable.

⁎*p*≤ .05; ⁎⁎⁎*p*≤ .001.

**TABLE 5:** Oppositional Dimensions and Their Associations With Parental History of Psychopathology

Oppositional dimensions	**Parental History of Psychopathology**					
Depression	Suicidality	History of ADHD Symptoms	Drinking	History of Conduct Symptoms	Imprisonment
Irritable	1.3 (1.1, 1.5)⁎⁎	1.2 (1.0, 1.4)⁎	1.0 (0.83, 10.2)	0.97 (0.76, 1.2)	1.0 (0.85, 1.2)	0.98 (0.73, 1.3)
Headstrong	1.1 (0.96, 1.3)	1.1 (0.90, 1.3)	1.3 (1.0, 1.6)⁎	1.1 (0.92, 1.5)	1.0 (0.86, 1.2)	1.0 (0.75, 1.3)
Hurtful	1.0 (0.94, 1.2)	1.1 (0.95, 1.2)	0.92 (0.77, 1.0)	1.0 (0.82, 1.2)	1.0 (0.91,1.2)	1.1 (0.92, 1.4)
Comparisons	Irr Head Hurt	Irr Head Hurt	Head Irr Hurt	Irr Head Hurt	Irr Head Hurt	Irr Head Hurt

Note: Values correspond to standardized odds ratios of multivariate logistic regression models (with the three dimensions as independent variables controlled for age and gender) and their 95% confidence interval in brackets for the oppositional dimensions. Comparison column shows contrasts between individual odds ratios, testing the null hypothesis that they do not differ, those with common underlining are not significantly different at the level of *p*<.05. ADHD = attention-deficit/hyperactivity disorder; Head = headstrong; Hurt = hurtful; Irr = irritable.

⁎*p* ≤.05; ⁎⁎*p* ≤.01.
